# Neonatal screening for congenital hypothyroidism: 28-year experience in the state of Minas Gerais, Brazil

**DOI:** 10.1016/j.jped.2024.12.007

**Published:** 2025-03-18

**Authors:** Nathalia Teixeira Palla Braga, Debora Patrícia da Silva Sousa Alves, Enrico Antônio Colosimo, Vera Maria Alves Dias, José Nélio Januário, Ivani Novato Silva

**Affiliations:** a Pediatric Endocrinology Service, Hospital das Clínicas, Universidade Federal de Minas Gerais, Brazil; b Department of Statistics, Universidade Federal de Minas Gerais, Brazil; c Center for Actions and Research in Diagnostic Support (NUPAD in Portuguese), Medicine Internal Department/Medical School, Universidade Federal de Minas Gerais, Brazil; d Pediatrics Department, Medical School, Universidade Federal de Minas Gerais, Brazil

**Keywords:** Congenital hypothyroidism, Neonatal screening, Incidence, Threshold

## Abstract

**Objective:**

The objective of this study was to determine the incidence of congenital hypothyroidism (CH) in Minas Gerais, Brazil, and evaluate the development of the Minas Gerais Neonatal Screening Program (PTN-MG) over the past 30 years.

**Method:**

This was a retrospective longitudinal cohort study since the implementation of neonatal screening for CH, in 1994. Bloodspots on filter paper are collected, between the third and fifth day of life, at primary healthcare units, with a TSH threshold of 10 mIU/L. The identification of an abnormal result triggers an active search for the child to confirm the diagnosis. The incidence of CH and its variation over the years, the percentage of permanent cases, and the age at sample collection and treatment initiation were analyzed.

**Results:**

The incidence of CH was 1:3,298 live births among 6,864,719 newborns screened, with no trend of change over the years (*p* = 0.08). The median age at sample collection decreased from 11 to 5 days (*p* < 0.01) and at treatment initiation from 88 to 16 days (*p* < 0.01). Among the confirmed patients, 77 % had permanent CH, thyroid dysgenesis accounted for 43.6 % of cases, gland-*in-situ* for 56.3 %.

**Conclusion:**

The incidence of CH has remained stable in Minas Gerais over the past 28 years. The PTN-MG is a public health program with an active monitoring and control sector that has shown significant improvements in its indicators since its implementation. The experience of the program has shown that rigorous monitoring and follow-up of infants have been an essential strategy for achieving satisfactory results.

## Introduction

Early detection and treatment of primary congenital hypothyroidism (CH) in newborns have virtually eradicated severe consequences in regions with neonatal screening since the 1970´s and 1980´s.^1^

Brazil implemented neonatal screening programs for CH in the 1990s, covering all states. The National Neonatal Screening Program (PNTN) recommends measuring neonatal thyroid-stimulating hormone (TSH) in a dried blood spot, with a threshold of 10 mIU/L.^2^ The Neonatal Screening Program of Minas Gerais (PTN-MG) was established in 1993 and has been covering the entire population of the state since 1994.

Despite its unequivocal importance, there is still a great discrepancy in the implementation and coverage of neonatal screening programs around the world. Severe intellectual deficits persist in areas without access to screening, affecting an estimated 70 % of newborns worldwide.^1^ According to data from the International Society for Neonatal Screening (ISNS), almost 100 % of babies born in European countries, the United States, Canada, and Australia have access to neonatal screening, whereas in Latin America, this percentage varies between 10 and 80 %, and in Africa, <2 % of newborns undergo testing.^3^^,^^4^

In the pre-screening period, when the disease was suspected due to its clinical manifestations, the reported incidence of CH was 1 case for every 6700 live births. Soon after the implementation of screening programs, there was an increase to approximately 1:3500.^5^^,^^6^ Over the last 20 years, an increase in the incidence of CH has been reported, mainly—but not exclusively—related to the greater number of mild cases without anatomical abnormalities in the thyroid, especially related to the reduction of TSH thresholds in screening programs.^7^, ^8^, ^9^, ^10^

There is no ideal cutoff point for screening programs, since it can vary according to geographic location, degree of iodine sufficiency, and even the particularities of the region's health system, and it should be individualized for the population.

Considering these issues, after 30 years of PTN-MG activity, the objective of this study was to determine the incidence of CH in MG, and evaluate the development of the program during this period, characterizing the screened population.

## Methods

A longitudinal and retrospective cohort study was carried out between January 1994 and November 2021, with data from the public neonatal screening program - PTN- MG - since its implementation.

The Research Ethics Committee of the Federal University of Minas Gerais (UFMG) approved the study (CAAE 50311321.1.0000.5149).

MG ranks fourth in geographic area (588,383 km^2^ and 853 municipalities) and second in population in Brazil, and is located in the Southeast region of Brazil. The program screens approximately 18,000 to 20,000 newborns per month. The PTN-MG covers the entire state.

The technical operation of the PTN-MG is carried out by the Núcleo de Ações e Pesquisa em Apoio Diagnóstico (NUPAD) [Center for Diagnostic Support Action and Research] of the Medical School of the Universidade Federal de Minas Gerais, under the management of the State Department of Health of Minas Gerais (SES-MG).

The PTN-MG adhered to the protocol recommended by the PNTN throughout the study period, measuring TSH (thyroid-stimulating hormone) in dried bloodspot (b-TSH) with a cut-off of 10 mIU/L. Bloodspots on filter paper (S&S 903®) are collected, between the third and fifth day of life, at primary healthcare units (90 %) or birth hospitals (10 %), from newborns who were not discharged by the fifth day due to relevant clinical conditions. There are 3744 collection points. Samples are sent via postal service to the referenced laboratory at NUPAD and results are typically released within 24 h of sample receipt. Given the vast size of MG, the average transportation time from sampling to arrival at the laboratory may vary, up to 5 days. B-TSH was assessed by fluoroimmunoassay method (GSP Neonatal hTSH, Turku, Finland).

The regional flow was inserted into the Brazilian Unified Health System (SUS) network and integrated with primary health care units (UBS) and birth hospitals, which are in contact with the Nupad monitoring and control sector.

**Figure 1:**
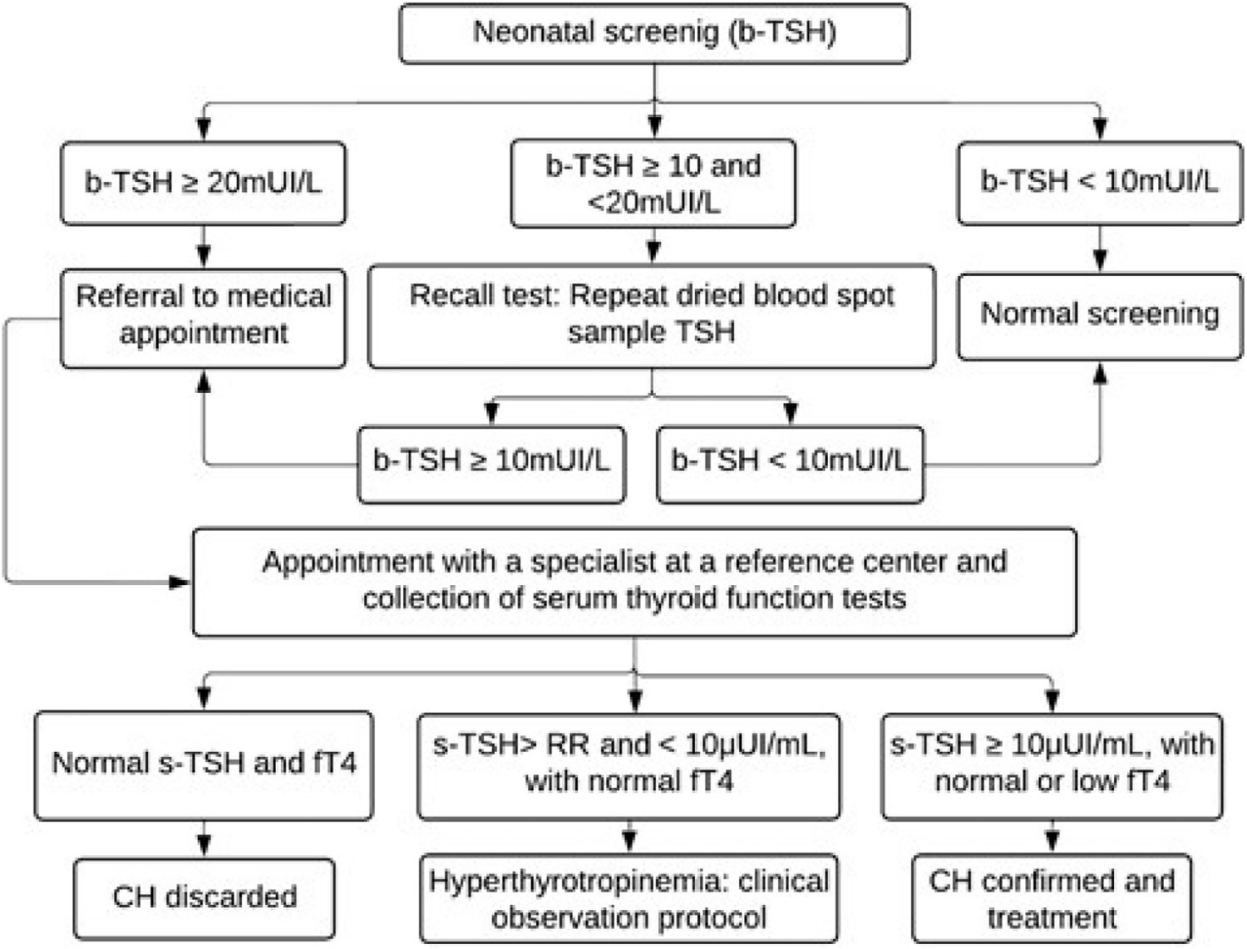
Flowchart for the diagnosis of congenital hypothyroidism based on neonatal screening in the Minas Gerais Neonatal Screening Program from 1994 to 2021. TSH, thyroid-stimulating hormone; b-TSH, filter-paper blood-spot TSH; s-TSH, serum TSH; FT4, free thyroxine; RR, reference range; CH, congenital hypothyroidism.

Children with TSH levels greater than or equal to 20 mIU/L were urgently called for medical appointments and diagnostic confirmation at the reference center. Those with borderline results (TSH between 10 and 20 mIU/L) underwent a new collection on filter paper (second sample) as soon as possible, and were called for appointment if the result remained greater than or equal to 10 mIU/L. The flowchart for diagnosing CH is shown in Figure 1.

Extremely premature infants (< 32 weeks of gestation or < 1500 *g*) and newborns with hemodynamic instability, who were at risk of false-negative screening, underwent serial collections, even if the first sample was normal (special protocols). Neonatal screening was repeated at 10 and 30 days of life. Newborns who received a blood transfusion before the third day of life underwent a repeat collection ten days after the transfusion.

The diagnosis was confirmed by measuring serum TSH (s-TSH – reference value [RV]: 0.69–8.55 μIU/mL) and free thyroxine (FT4 - RV: 0.89–1.76 ng/dL) levels by chemiluminescence (ICMA). The criteria used to confirm cases and initiate treatment were s-TSH ≥10 μIU/mL and normal or low FT4 levels, or borderline s-TSH (above the reference value but below 10 μIU/mL) associated with low FT4. Patients diagnosed by this last criterion were evaluated for the possibility of central CH.

Children with hyperthyrotropinemia, defined as borderline s-TSH (above the reference value but < 10 µUI/mL) associated with normal FT4, were followed up according to the clinical observation protocol: undergoing strict clinical and laboratory monitoring without drug therapy. Treatment was indicated in the case of a confirmed diagnosis. Treatment with levothyroxine (Levoxyl) was initiated in all confirmed cases. The first consultations were conducted by the PTN-MG team at the reference center, Hospital das Clínicas da Universidade Federal de Minas Gerais (HC-UFMG). Subsequent follow-up, including quarterly assessments up to 3 years of age, was carried out collaboratively at both the reference center and in the patient's municipality of residence –at Primary Health Care Facilities (UBS)– by registered physicians and guided by endocrinologists from PTN-MG.

After the age of 3, children undergoing treatment were subjected to laboratory and imaging tests to determine the etiology and to differentiate between permanent and transient cases, following discontinuation of levothyroxine (Levoxyl) for 4–6 wk. During the periods in which imaging tests were not available, patients using low doses of levothyroxine (Levoxyl) had their treatment temporarily suspended to assess the possibility of the condition being transient. Children who continued to require treatment with levothyroxine (Levoxyl) after reassessment at the age of 3 years comprised the permanent CH group and were monitored by the PTN-MG until the age of 18 years.

Imaging tests were performed in the Radiology Department of HC-UFMG. Ultrasounds of the thyroid gland were performed by a team of pediatric radiologists. Thyroid scintigraphy was performed with technetium 99 m or iodine 131, according to availability at the time. The perchlorate test was conducted whenever available.

The etiology of permanent CH was assessed based on the results of ultrasound, scintigraphy, and thyroglobulin levels. Cases with anatomical alterations identified in imaging tests (hypoplasia, athyreosis, hemiagenesis, and ectopia) were classified as dysgenesis. The remaining cases were classified as gland*-in-situ* (GIS) and further subdivided based on their characteristics. Patients with goiter associated with the absence of radioisotope uptake on scintigraphy were diagnosed with dyshormonogenesis due to a sodium-iodide symporter (NIS) defect. Those with goiter, increased radioisotope uptake, and undetectable thyroglobulin (Tg) were diagnosed as dyshormonogenesis due to Tg defect. The remaining cases were divided into two groups: patients with goiter and those with normal-sized GIS.

### Statistical analysis

The incidence of CH was calculated based on the ratio between the number of confirmed cases and the total number of newborns screened in the entire period and annually.

The percentage of transient cases was calculated by subtracting the number of permanent cases from the total diagnoses assessed in patients over 3 years of age. For this calculation, only patients who remained under follow-up until this age were considered. Patients who died, migrated, or left the healthcare system were not included.

Confirmed cases with FT4 below the reference value upon diagnosis were classified as decompensated CH.

The age of newborns at collection of the first blood-spot sample and upon diagnosis, at the start of CH treatment, as well as their variations over time, at 5-year intervals, were analyzed.

The program coverage was determined by the ratio between the number of live births recorded by the Unified Health System database—DATASUS^11^ and the total number of newborns screened during the period.

The Kolmogorov-Smirnov or Shapiro-Wilk tests were applied to assess the normality of the distribution of quantitative variables. Continuous variables were characterized by medians, minimum and maximum values, and –if applicable – percentiles of interest. Comparisons between these variables were performed using the Kruskal- Wallis test, with Bonferroni post-hoc test. Categorical variables were represented by their absolute and percentage values and compared using Pearson's chi-square test. Linear regression model was built to identify associations between variables. The analyses were performed using R 4.1.2 and IBM SPSS Statistics 26 software. A p-value ≤ 0.05 was considered statistically significant.

## Results

Since the program was implemented in 1994 until November 2021, a total of 6,864,719 newborns were screened by the PTN-MG. In this period, 1996 CH cases were confirmed.

The incidence of CH was calculated for the period from 1997 to 2021, as the years 1994 to 1996 were excluded due to the inability to recover all diagnosed cases of CH. During this period, 6,144,415 newborns were screened, and 1863 were diagnosed. The incidence of CH in the period from 1997 to 2021 was 1 case for every 3298 live births – or 3.03 cases for every 10,000 live births.

**Figure 2:**
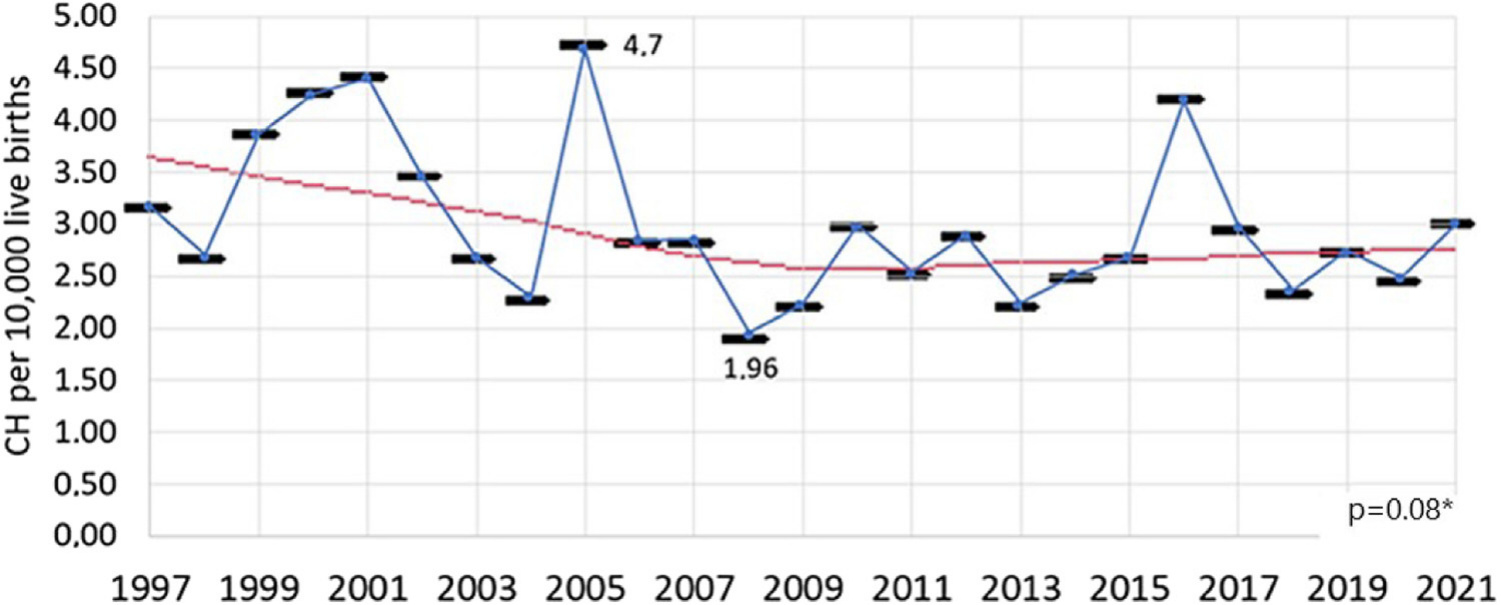
Incidence of congenital hypothyroidism between 1997 and 2021 in Minas Gerais (number of cases per 10,000 live births). CH, congenital hypothyroidism. *Linear regression.

The incidence varied over the years studied (*p* < 0.01), with no trend of increase or decrease observed in the linear regression analysis (*p* = 0.08) throughout the entire period (Figure 2).

The calculation of permanent CH cases was made between 1998 and 2016, due to inconsistent data between 1994 and 1997 available in DATASUS. A total of 1242 (77 %) patients born until 2016 and reassessed until 2019, had permanent CH. In the linear regression analysis, no trend of change in the number of cases of permanent hypothyroidism was identified in the period (*p* = 0.24) and, consequently, in the number of transient cases.

Forty-three percent of newborns had decompensated CH. There were significantly more occurrences of decompensated cases in 2007 and 2011, and compensated cases between 1994 and 2001 (*p* < 0.01); however, in the linear regression analysis, no trend of modification was identified over the years (*p* = 0.25).

**Figure 3:**
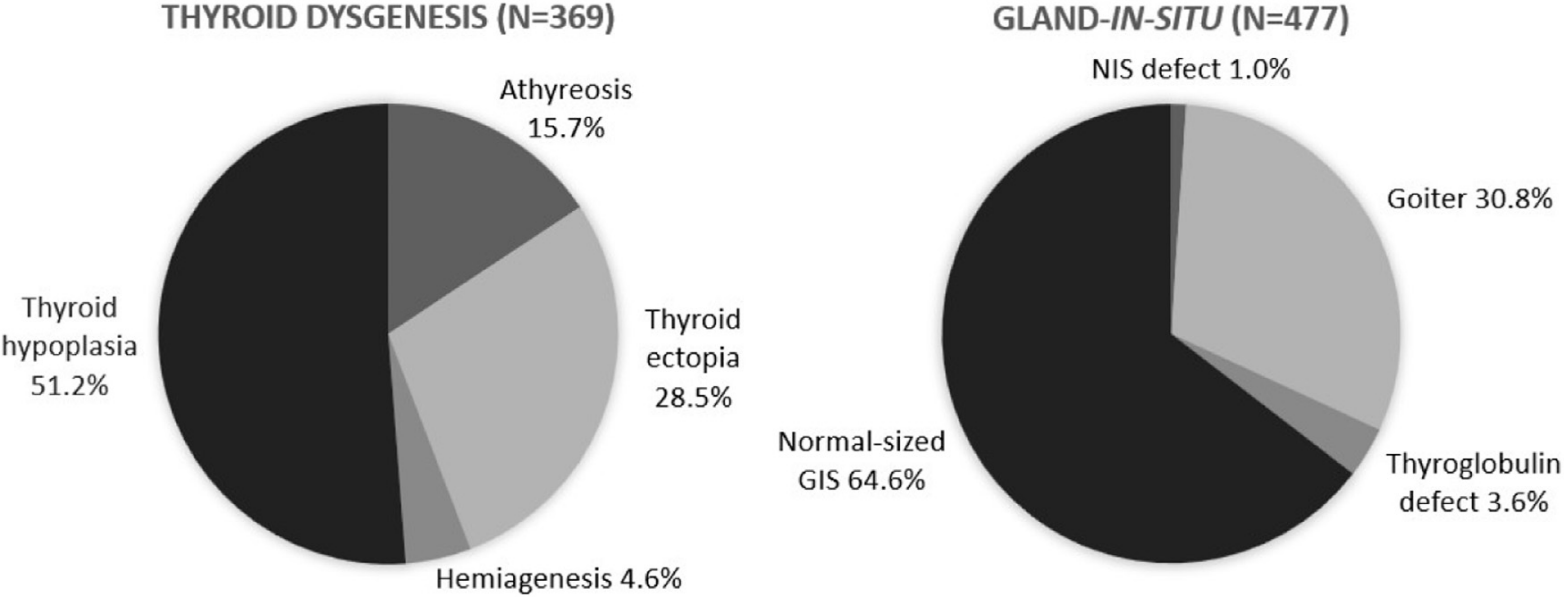
Distribution of cases of thyroid dysgenesis and gland-*in-situ* in congenital hypothyroidism patients between 1994 and 2019 in Minas Gerais.

The etiological evaluation of CH by imaging exams was carried out in 846 (68 %) patients with permanent CH. Thyroid dysgenesis and GIS accounted for 43.6 % and 56.3 % of cases, respectively. There was a significant increase in the proportion of all forms of dysgenesis and a decrease in GIS cases, particularly due to a reduction in the number of normal-sized GIS cases (*p* < 0.01) over the years. The distribution of cases of dysgenesis and GIS is illustrated in Figure 3.

The median age at which the first blood-spot sample was collected significantly decreased between the study periods, from 11 days in the first years (1994–1996) to 5 days since 2007 (*p* < 0.01)

**Figure 4:**
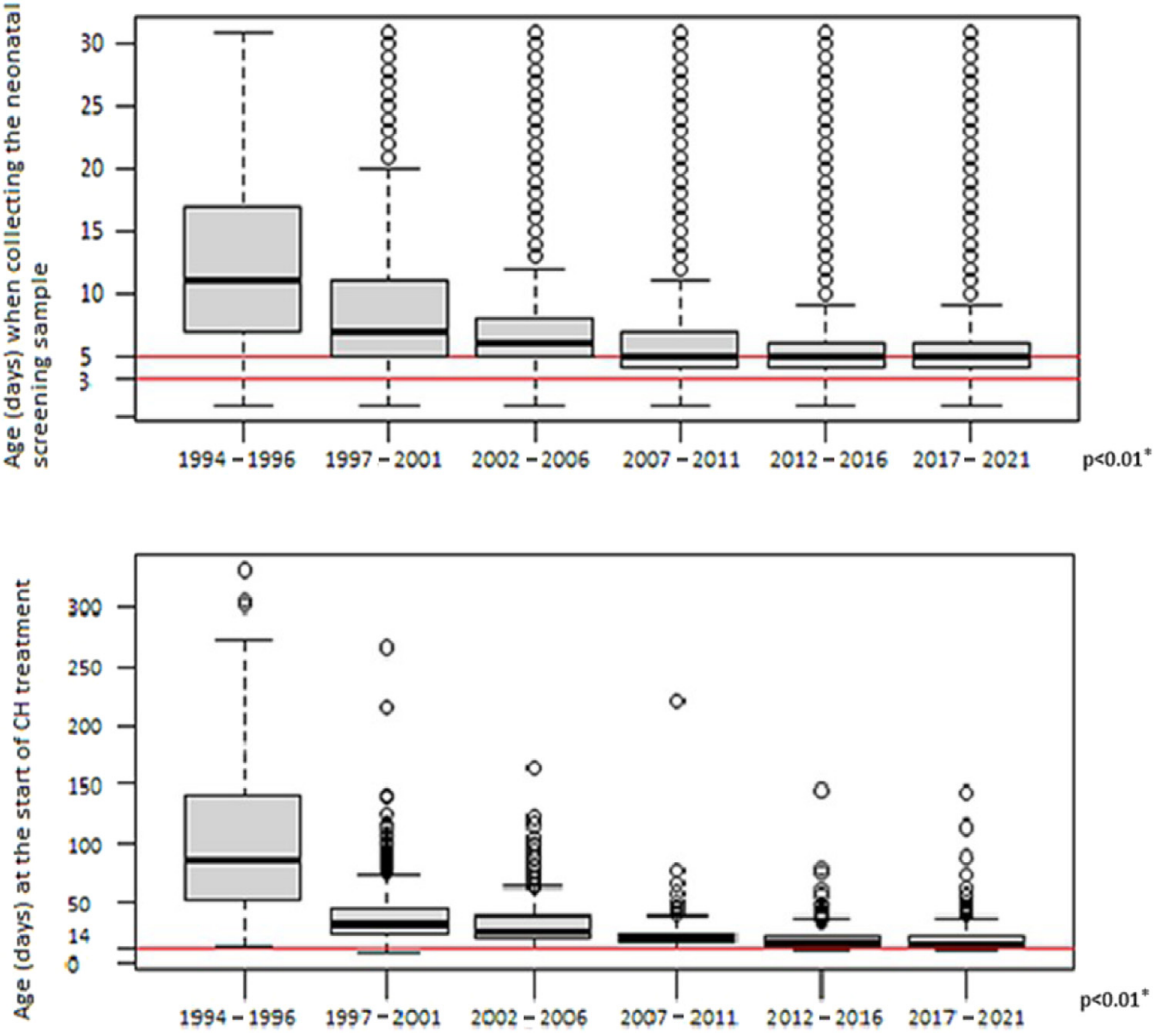
Age at neonatal first blood-spot sample collection and initiation of treatment for congenital hypothyroidism between 1994 and 2021 in Minas Gerais. CH, congenital hypothyroidism. *Kruskal–Wallis.

The median age at which treatment was initiated significantly decreased from 88 days in the first years (1994 to 1996) to 16 days between 2017 and 2021 (*p* < 0.01). Figure 4 shows the variation in age of blood-spot collection and treatment initiation over the years.

The coverage of the PTN-MG in the territory was 91.5 %, and reached its maximum value –95 % of the population – in the years 2000, 2006, and 2007. This percentage has been falling significantly (*p* < 0.01) and reached 88 % in the last 2 years evaluated.

## Discussion

From 1997 to 2021, the incidence of CH in MG was 1 case for every 3298 live births, remaining stable over a 28-year period. The latest consensus on the disease indicates a mean incidence between 1:2000 and 1:3000 live births, but with great variability among populations and screening programs.^1^

In Brazil, the incidence of CH varies widely from 2595 to 4795.^2^ Even in states employing the Ministry of Health protocol, such as the PTN-MG, variations in incidence were reported, such as 1:2319 (*n* = 4,188,792) in the South region^12^ and 1:4632 (*n* = 254,782) in the North of the country.^13^ Since the country is remarkably diverse, the ethnic composition of the populations may be responsible for these differences. Some states have reported an increased incidence of the disease after implementing protocols with greater sensitivity and cutoff points ranging from 4.5 to 9.0 mIU/L: in Rio de Janeiro, 1:1101^14^; in Santa Catarina, 1:1560^15^ in Sergipe, 1:950^16^ and in Rio Grande do Sul, 1:2377.^17^

The increased incidence of CH reported in the past 20 years around the world^1^^,^^5^^,^^6^ has not just one explanation. The incidence of CH has remained stable in some regions, such as Finland^18^ and Canada,^8^ even with changes in screening protocols and ethnic population. On the other hand, in some regions, such as New Zealand, New York, Japan and Ireland, the incidence has practically doubled.^19^, ^20^, ^21^

The increased survival of premature and low-birth-weight newborns may contribute to the rise in transient CH cases detected through neonatal screening. Immaturity of the hypothalamic-pituitary-thyroid axis, clinical intercurrent events, and use of medications that may interfere with thyroid function are more frequent in this group of children.^19^^,^^20^ Advanced maternal age may also be implicated in an increased number of CH cases, due to the greater risk of genetic mutations, in addition to gestational complications, which contribute to more premature births.^19^^,^^20^ Furthermore, migration-driven ethnic changes, particularly in Hispanic and Asian descent populations, which have a higher incidence of CH, may justify this rise.^19^^,^^22^

Nonetheless, using lower TSH cutoff points in screening protocols is the factor most strongly associated with a higher incidence of CH.^5^^,^^7^^,^^10^^,^^23^ Increased sensitivity in screening programs has been associated with a higher proportion of mild, transient diseases or cases caused by dyshormonogenesis.^8^^,^^9^^,^^24^ There is ongoing debate regarding the benefits of treating mild CH, as the outcomes from the treatment of moderate and severe cases may not be directly applicable.^24^

The percentage of transient CH cases, which was 23 % and remained constant throughout the study period, is strongly associated with the cutoff point used in the screening programs and varies greatly among regions, ranging from 3.6 % to as high as 40 % of cases.^25^, ^26^, ^27^ The stability of this proportion is likely explained by the fact that the b-TSH cutoff point remained unchanged during the period evaluated in the present study.

An increase was observed in the percentage of thyroid gland dysgenesis and a decrease in cases of normal-sized GIS over the years, which may reflect an improved ability to identify these cases. The proportion of dysgenesis—43.6 % in the current cohort—was lower than the approximately 85 % typically reported in the literature. Conversely, the proportion of GIS was higher, at 56.4 %, compared to the reported 15 %.^1^ These elevated percentages of GIS observed in MG are comparable to those reported in regions where the TSH screening threshold was reduced.^8^^,^^9^^,^^24^^,^^28^ It can be hypothesized that the relatively low cutoff value of 10 mIU/L, historically used in the PTN-MG, compared to that initially used in most international protocols (20–40 mIU/L), when these classic proportions for CH etiologies were identified, could explain this finding. However, in southern Brazil, in the state of Paraná, using the same cutoff point as the PTN-MG, 65 % of cases of permanent CH secondary to dysgenesis were reported,^12^ whereas in Santa Catarina, 68.75 % were secondary to dyshormonogenesis.^15^ Therefore, it is not possible to rule out that the findings in MG are related to ethnic differences in its population, because the occurrence of dyshormonogenesis has a strong genetic influence.

Since its implementation, the PTN-MG has had excellent coverage, reaching 95 % of live births by 2007. The program is primarily targeted at the SUS population, and covers all 853 municipalities of the state. The average coverage in Brazil was 82.53 % in 2020, with high variability among Brazilian states.^29^ The provision of tests with expanded neonatal screening panels in the private healthcare system, along with the increase in the number of people insured by health plans in MG, may have driven the rise in screenings outside the public system, which could explain the decline in coverage observed in recent years. However, screening in the private sector fails to meet the notification and newborn's active search requirements, potentially impacting the prognosis of affected children. The absence of official data on neonatal screening in the private sector makes this assessment difficult.

The efficiency of a screening program for CH is closely linked to the time taken for transporting and processing samples, as well as to locating and providing immediate care to children, thereby preventing mental retardation associated with late diagnosis.

The timing for collecting the first blood-spot sample has improved and met targets in PTN-MG over the years. The strategic axis of Humanized Care for Pregnancy, Labor, Birth and Newborns in the SUS, which emphasizes the adoption of the 5th Day of Comprehensive Health, has been critical to this success. In 2020, 58.06 % of newborns in Brazil underwent their first neonatal screening test by day 5, a figure that could be enhanced to further optimize the initiation of early treatment.^29^

The age at which treatment begins has also been reduced. The recent stabilization of this indicator may be related to challenges that need to be addressed to achieve the goal of starting treatment within the first 2 weeks of life, as recommended.^1^ Neonatal screening in MG presents a significant challenge for public health due to the state's large and diverse territory, which is exemplified by transportation issues for samples in remote regions.

In Brazil, a country marked by great diversity, the median age for medical evaluation following altered screening for CH was 35 days in 2020.^29^ A recent report shows the existence of inequalities of access according to the region of residence, income, and having health insurance, and highlights the need to develop strategies to promote universal access and equity.^30^ These inequalities may become more pronounced in exceptional situations, such as during the COVID-19 pandemic. Recent data suggest an increase in the age at first consultation during this period.^29^ However, data from PTN-MG showed no impact on treatment coverage or the age at treatment initiation, which can be attributed to the dedication and proactive monitoring efforts of the healthcare professionals involved.

The experience of PTN-MG has shown that rigorous monitoring and follow-up of newborns in UBS or birth hospitals have been an essential strategy for achieving satisfactory results. Ongoing screening program monitoring and data analysis are essential for identifying necessary interventions and improvements. Continuous population awareness of the benefits of proper neonatal screening is also crucial.

In conclusion, PTN-MG has provided free access to neonatal screening tests for CH to the entire population since 1994, in a satisfactory and successful manner. From its early years until the end of 2021, the incidence has remained stable at 1 case per 3298 live births. These data show that it is possible to maintain an excellent screening program within the public health system, benefiting all children.

## Funding

This research received no external funding.

## Authors’ contributions

Conceptualization, methodology N.T.P.B., V.M.A.D., and I.N.S.; investigation, N.T.P.B. and D.P.S.S.A.; formal analysis, N.T.P.B. and E.A.C.; original draft preparation, N.T.P.B.; writing—review and editing, I.N.S.; V.M.A.D., and J.N.J.; supervision, I.N.S. All authors have read and agreed to the published version of the manuscript.

## Conflicts of interest

The authors declare no conflicts of interest.
